# Rapid streptococcal pharyngitis testing and antibiotic prescribing before and during the coronavirus disease 2019 (COVID-19) pandemic

**DOI:** 10.1017/ash.2022.222

**Published:** 2022-05-10

**Authors:** Allan M. Seibert, Edward Stenehjem, Anthony Wallin, Park Willis, Kim Brunisholz, Naresh Kumar, Valoree Stanfield, Nora Fino, Daniel J. Shapiro, Adam Hersh

**Affiliations:** 1 Office of Research, Intermountain Healthcare, Salt Lake City, Utah; 2 Division of Infectious Diseases and Clinical Epidemiology, Intermountain Healthcare, Salt Lake City, Utah; 3 Intermountain Urgent Care, Intermountain Healthcare, Salt Lake City, Utah; 4 Healthcare Delivery Institute Intermountain Healthcare, Salt Lake City, Utah; 5 Division of Epidemiology, Department of Internal Medicine, University of Utah, Salt Lake City, Utah; 6 Division of Emergency Medicine, Boston Children’s Hospital, Boston, Massachusetts; 7 Division of Pediatric Infectious Diseases, Department of Pediatrics, University of Utah, Salt Lake City, Utah

*To the Editor—*Pharyngitis is one of the most common conditions leading to inappropriate antibiotic prescriptions.^1,2^ Rapid group A streptococcal (GAS) testing remains a key component of care to guide appropriate antibiotic prescribing for patients with acute pharyngitis.^3,4^ The percentage of pharyngitis encounters prescribed an antibiotic and that underwent GAS testing is also a key measure of the Healthcare Effectiveness Data and Information Set.^5^ In the initial months of the coronavirus disease 2019 (COVID-19) pandemic when personal protective equipment (PPE) was constrained, Intermountain Healthcare recommended limiting rapid GAS testing in urgent care clinics to preserve PPE. We describe our experience with pharyngitis encounters and the impact of temporarily reducing GAS testing on antibiotic prescribing for pharyngitis before and during the COVID-19 pandemic.

We performed a retrospective cohort study of pharyngitis encounters within the Intermountain Healthcare urgent-care network from July 2018 through August 2021. Intermountain Healthcare is a nonprofit, vertically integrated healthcare delivery system with 38 urgent-care clinics in Utah. Moreover, 32 of these clinics provide care for patients of all ages and 6 provide care exclusively to children aged <18 years. All provide only in-person care and do not provide telehealth services. On April 1, 2020, urgent-care leadership recommended limiting rapid GAS testing to children aged ≤15 years and empirically treating most adults where GAS was considered likely. More specific guidance was not offered. In late May 2020, urgent-care leadership instructed clinicians to resume routine testing practices, which strongly encourage testing when prescribing antibiotics. We identified all urgent-care encounters associated with a primary diagnosis of pharyngitis using *International Classification of Diseases, Tenth Edition, Clinical Modification* (ICD-10) codes (Supplementary Material) and a validated methodology.^6^ Encounters were assessed for antibiotic prescriptions ordered through the electronic health record (EHR) and the use of point-of-care rapid GAS tests. Pharyngitis encounters were analyzed monthly by assessing the percentage of encounters associated with an antibiotic prescription regardless of testing and the percentage of encounters associated with an antibiotic prescription when a GAS test was or was not performed. Three periods relating to COVID-19 and GAS testing recommendations were examined: prepandemic (July 2018–March 2020), pandemic onset (April 2020–June 2020), and the pandemic (July 2020–August 2021).

In total, 115,558 encounters for pharyngitis were identified. Most patients were white (92.7%) and 59.2% were female. The average age was 25.0 years: 74,107 (64.1%) were aged ≥18 years and 38,776 (33.6%) were aged 3–18 years. During the prepandemic period, the monthly percentage of pharyngitis encounters for which rapid GAS testing was performed, regardless of an antibiotic prescription, was consistently near 90%. The average percentage of monthly pharyngitis encounters prescribed an antibiotic that also underwent GAS testing was 90.4%. Following the recommendation to limit testing, the monthly percentage of pharyngitis encounters for which rapid GAS testing was performed sharply declined from 84.2% in March 2020 to 37.9% in April 2020. The average monthly percentage of pharyngitis encounters in urgent care in which an antibiotic was prescribed increased from 38.9% to 50.6% during this pandemic onset period. In the prepandemic period, an average of 9.6% of pharyngitis encounters were prescribed an antibiotic without GAS testing, and this rose to 70.2% during the pandemic onset period. The monthly percentage of pharyngitis encounters in urgent care for which rapid GAS testing was performed returned to levels ≥80% by July 2020 as routine testing practices were restored. The average percentage of monthly pharyngitis encounters prescribed an antibiotic that also underwent GAS testing rose to 87.3% during the pandemic period. Additional patient demographics and testing characteristics are available in Supplementary Tables 1 and 2.

As rapid GAS testing volumes decreased in the urgent-care centers of our healthcare network due to limited PPE during the initial months of the COVID-19 pandemic, we observed a simultaneous relative increase of >30% in antibiotic prescribing for pharyngitis driven by an increase in antibiotic treatment for pharyngitis without testing (Fig. 1). We observed this trend across all age groups. Prior studies have consistently demonstrated the poor sensitivity and specificity of clinical criteria alone to diagnose GAS pharyngitis in children and adults.^7,8^

**Fig. 1:**
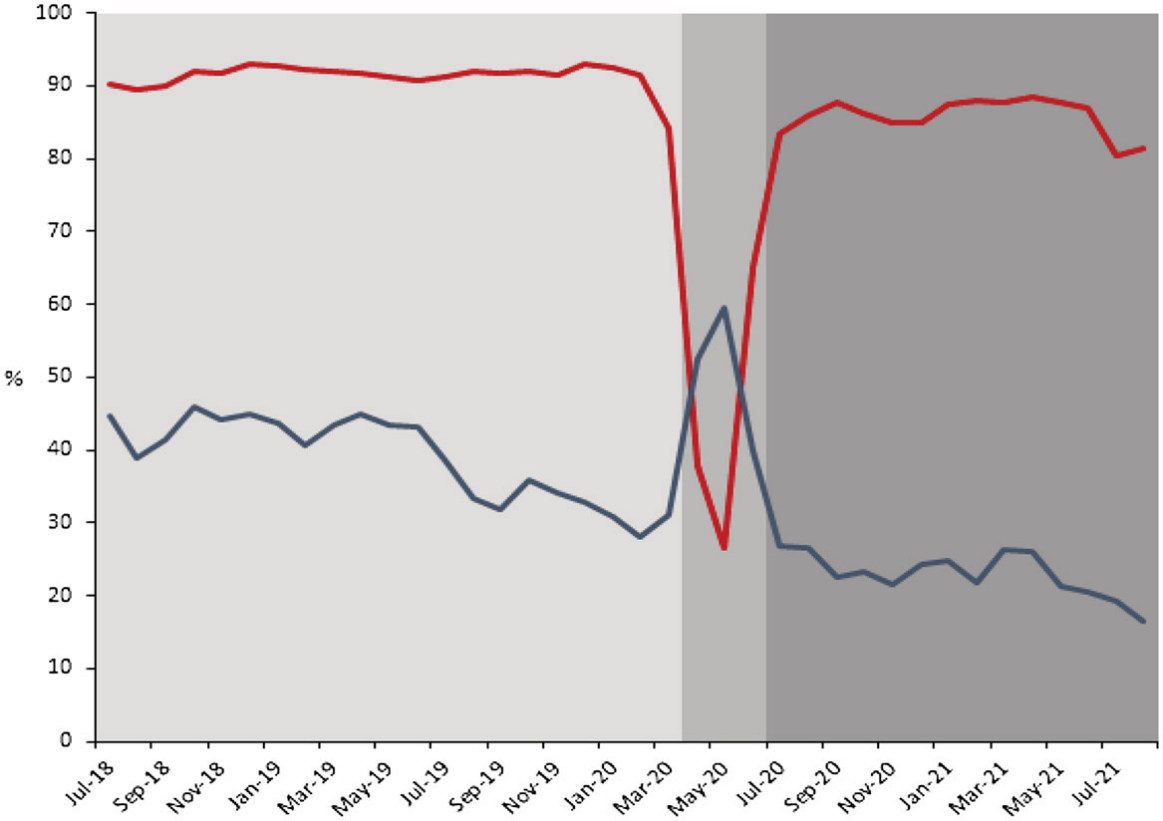
Monthly Pharyngitis GAS testing (red line) and antibiotic prescribing rates (blue line) July 2018–August 2021. Each time period examined is indicated in gray shading. Limited testing was associated with a concomitant increase in antibiotic prescribing for pharyngitis. Antibiotic prescribing returned to prepandemic levels and continued to decline as testing volumes returned to prepandemic levels.

Our study had several limitations. We did not explicitly monitor the extent to which appropriate testing criteria were used nor did we examine the clinical characteristics of patients presenting with pharyngitis. The prevalence of GAS could have increased during the pandemic onset. However, the percentage of positive point-of-care GAS tests for pharyngitis encounters was lower during the period of limited GAS testing when compared to prepandemic levels (18.4% vs 24.6%, respectively). An abrupt rise in GAS infections is also unlikely because the incidence of other respiratory infections in general declined with social-distancing measures. The observed decrease in antibiotic prescribing for pharyngitis encounters to levels below those preceding the pandemic onset may be explained in part by the simultaneous introduction of an urgent-care–based antibiotic stewardship program intervention between July 2019 and June 2020.^9^ The percentage of pharyngitis encounters prescribed an antibiotic associated with a negative GAS test transiently increased, but the main driver of increased antibiotic prescriptions overall during this period were pharyngitis encounters in which no GAS test was performed. In conclusion, our experience with temporarily limiting rapid testing highlights its value in guiding the evaluation and appropriate antibiotic therapy in patients suspected of having GAS pharyngitis.

## Supplementary material

For supplementary material accompanying this paper visit https://doi.org/10.1017/ash.2022.222. click here to view supplementary material
