# Supplementary material for: Rapid streptococcal pharyngitis testing and antibiotic prescribing before and during the coronavirus disease 2019 (COVID-19) pandemic
Source: Antimicrob Steward Healthc Epidemiol. 2022 May 10;2(1):e80. doi: 10.1017/ash.2022.222 (PMC9726542; doi:10.1017/ash.2022.222)
Supplement: Supplementary file 1 [file S2732494X22002224sup001.docx]

**Table 1**

| *Characteristic* | *Overall* | *July 2018 - March 2020* | *April 2020 - June 2020* | *July 2020 - August 2021* |
| --- | --- | --- | --- | --- |
| Pharyngitis encounters, N | N = 115,558 | n = 103,454 | n = 3,773 | n = 8,331 |
|  |  |  |  |  |
| Received Antibiotic, N (%) |  |  |  |  |
| No | 71,519 (61.9%) | 63,286 (61.2%) | 1,891 (50.1%) | 6,342 (76.1%) |
| Yes | 44,039 (38.1%) | 40,168 (38.8%) | 1,882 (49.9%) | 1,989 (23.9%) |
| Female, N (%) |  |  |  |  |
| No | 47,196 (40.8%) | 42,410 (41.0%) | 1,538 (40.8%) | 3,248 (39.0%) |
| Yes | 68,362 (59.2%) | 61,044 (59.0%) | 2,235 (59.2%) | 5,083 (61.0%) |
| Patient Age, years | 25.0 (16.2) | 24.8 (16.3) | 27.5 (15.3) | 26.3 (15.1) |
| Age Group, N (%) |  |  |  |  |
| 0-3 years | 2,675 (2.3%) | 2,462 (2.4%) | 79 (2.1%) | 134 (1.6%) |
| 3-18 years | 38,776 (33.6%) | 36,040 (34.8%) | 720 (19.1%) | 2,016 (24.2%) |
| 18+ years | 74,107 (64.1%) | 64,952 (62.8%) | 2,974 (78.8%) | 6,181 (74.2%) |
| White Race, N (%) |  |  |  |  |
| No | 8,478 (7.3%) | 7,524 (7.3%) | 328 (8.7%) | 626 (7.5%) |
| Yes | 107,070 (92.7%) | 95,920 (92.7%) | 3,445 (91.3%) | 7,705 (92.5%) |
| Clinic Type, N (%) |  |  |  |  |
| InstaCare^1^ | 105,900 (91.6%) | 94,330 (91.2%) | 3,627 (96.1%) | 7,943 (95.3%) |
| KidsCare^1^ | 9,658 (8.4%) | 9,124 (8.8%) | 146 (3.9%) | 388 (4.7%) |

^1^InstaCare is an urgent care clinic which provides care for patients of all ages while KidsCare sites are urgent care clinics which provide care exclusively to children. These are predominantly staffed by physicians. KidsCare is exclusively staffed by pediatricians.

**Table 2**

| *Characteristic* | *July 2018 - March 2020* | *April 2020 - June 2020* | *July 2020 - August 2021* |
| --- | --- | --- | --- |
| Average monthly percentage of pharyngitis encounters prescribed an antibiotic | 38.9% | 50.6% | 23.0% |
| Average monthly percentage of pharyngitis encounters not tested for GAS^2^ and prescribed an antibiotic | 42.2% | 63.2% | 20.9% |
| Average monthly percentage of pharyngitis encounters negative for GAS and prescribed an antibiotic | 18.7% | 17.7% | 13.1% |
| Average monthly percentage of pharyngitis encounters positive for GAS and prescribed an antibiotic | 99.4% | 98.1% | 99.0% |

**^2^**GAS: Rapid Group A Streptococcal testing
 **†ICD10 Codes for Pharyngitis Encounters**
J02.8 – Acute pharyngitis due to other specified organisms
J02.9 – Acute pharyngitis, unspecified
J03.80 – Acute tonsillitis due to other specified organisms
J03.90 – Acute tonsillitis, unspecified
J03.91 – Acute recurrent tonsillitis, unspecified
J06.0 – Acute laryngopharyngitis
J02.0 – Streptococcal pharyngitis
J03.00 – Acute streptococcal tonsillitis, unspecified
J03.01 – Acute recurrent streptococcal tonsillitis
